# Childhood trauma and current depression among Chinese university students: a moderated mediation model of cognitive emotion regulation strategies and neuroticism

**DOI:** 10.1186/s12888-021-03673-6

**Published:** 2022-02-07

**Authors:** Qianqian Chu, Xiang Wang, Rui Yao, Jie Fan, Ya Li, Fei Nie, Lifeng Wang, Qiuping Tang

**Affiliations:** 1 Department of Clinical Psychology, The Third Xiangya Hospital of Central South University, Changsha, Hunan 410013 China; 2 Medical Psychological Center, The Second Xiangya Hospital of Central South University, Changsha, Hunan 41000 China; 3 Center for Psychological Development and Service, Hunan University of Chinese Medicine, Hunan 410208 Changsha, China; 4 School of Nursing, Hunan University of Chinese Medicine, Changsha, Hunan 410208 China

**Keywords:** Childhood trauma (CT), Adaptive / maladaptive cognitive emotion regulation strategies, Neuroticism, Depression, Moderated mediation model

## Abstract

**Background:**

Childhood trauma (CT) is considered as a highly risk factor for depression. Although the pathway of CT to depression, especially the mediating or moderating effects of cognitive emotion regulation strategies (CERS) or neuroticism, have investigated by several studies, the results were inconsistent and there is a paucity of full models among these interactive factors. This study aims to examine the relationships among CT, adaptive / maladaptive CERS, neuroticism, and current depression symptoms in university students.

**Methods:**

We recruited 3009 freshman of 2019, aged averagely 18.00 (SD = 0.772) years, from universities in Hunan province in 2019. A moderated mediation model was built to examine the relationships among CT, CERS, neuroticism, and current depression using the SPSS PROCESS 3.5 macro. We conducted bootstrapping of regression estimates with 5000 samples and 95% confidence interval.

**Results:**

Results revealed that the significant mediating effects of adaptive CERS (*β* = 0.012; 95% CI: 0.006 to 0.018) and maladaptive CERS (*β* = 0.028; 95% CI: 0.016 to 0.040) between CT and depression were observed, accounting for 5.69% and 13.52% of the total effect respectively. Then, moderated mediation analyses results showed that neuroticism simultaneously moderated the direct effect of CT on current depression (*β* = 0.035; 95% CI: 0.001 to 0.009), and the indirect effects of CT on current depression through adaptive CERS (adaptive CERS – current depression: *β* = − 0.034; 95% CI: − 0.007 to − 0.001) and maladaptive CERS (maladaptive CERS – current depression: *β* = 0.157; 95% CI: 0.017 to 0.025). However, the moderating effects of neuroticism in the indirect paths from CT to adaptive CERS (*β* = 0.037; 95% CI: 0.000 to 0.014) and maladaptive CERS (*β* = − 0.001; 95% CI: − 0.006 to 0.005) were not significant.

**Conclusions:**

This study provides powerful evidences through a large university students sample for the mediating role of adaptive / maladaptive CERS and the moderating role of neuroticism between CT and current depression. This manifests that cognitive emotion regulation may be a vital factor for people who suffered from CT and current depression. Furthermore, the influence of neuroticism in this process cannot be ignored.

**Supplementary Information:**

The online version contains supplementary material available at 10.1186/s12888-021-03673-6.

## Background

### CT and depression

According to the World Health Organization (WHO), more than one third of the world’s population has experienced childhood trauma (CT). It is estimated that about one in five women and one out of 13 men have suffered from sexual abuse in childhood, and about 25% of adults have undergone physical abuse in childhood. In addition, patients with mental disorders, 28.9% of them reported having experienced CT, and the effects of CT may persist throughout the course of life [1]. The CT was considered as a particularly potent risk factor for the onset, symptomatic severity, and course of depression, which has been demonstrated by various previous studies [2, 3]. Depression is a major human blight, resulting in tremendous personal and social burden. Furthermore, meta-analytic evidence has revealed that experiencing any type of maltreatment may increase more than a two-fold risk for depression in adulthood [4]. The greater childhood adversity is related to more severe depression [5], more chronic depression [6], and a longer time to remission [7].

The Vulnerability-stress model [8] provides theoretical support for the close connection between childhood trauma and depression. In the study of the etiology of depression, researchers have found that an individual suffering from negative life events is prone to depression. However, when facing the same circumstance, individuals react differently: some people would experience depression, while others do not. This phenomenon makes people realize that there are certain variables that determine stress conditions who will be depressed. Researchers have also begun to use vulnerability to study the etiology of depression. Studies have shown that depression may occur after exposure to acute or chronic life stress, especially in people who have experienced childhood trauma exposure [9].

In the large number of existing studies on childhood trauma and depression, we can find that the factors of affecting the relationship between childhood trauma and depression can be divided into two categories: psychosocial and neurobiology. The psychosocial factors include cognition [10, 11], emotion regulation [12], and personality factors [13] etc. Although previous studies have established the basic relationship between childhood trauma and depression, the specific mechanism underlying CT and depression is still unclear.

### Psychological vulnerability affecting the relationship between CT and current depression

#### Cognitive vulnerability

Beck’s Cognitive Theory [14] pointed that cognition is a crucial influence factor in the occurrence and development of depression. Cognitive emotion regulation strategies (CERS) are the cognitive response of an individual consciously or unconsciously trying to change the size and / or type of their own emotional experience and the event itself when faced with an emotion-eliciting events [15]. Garnefski et al. [16] divided CERS into two categories: adaptive and maladaptive. A meta-analysis of emotion regulation strategies shows that compared with other disorders, emotion-related disorders are more closely related to emotion regulation strategies, and compared with adaptive strategies, maladaptive strategies are more closely and more consistent with psychopathology [17]. Cognitive theories put maladaptive appraisal processes at the core of depression and anxiety [18]. Recently, many studies had revealed that higher depression was related to more use of maladaptive CERS, and less use of adaptive CERS (e.g. positive reappraisal) [19, 20]. Furthermore, failure in regulating the emotion has been found to be closely related to the occurrence of psychological problems [21].

In addition, early adverse experiences can lead to the formation of latent depressive cognition schema, which affects the emotions, behaviors and thinking patterns of depressive patients [22]. For instance, physical abuse in childhood are related to schemas of danger and distrust, while experiencing emotional neglect is related to schemas of value and belonging [23]. CT may interfere with the healthy development of emotional regulation. Some researchers believed that acquiring appropriate emotion regulation skills can be disrupted by repeated interpersonal trauma between the caregiver and the child [24, 25]. Individuals who experienced CT, often struggle to understand their emotions, because they haven’t received effective feedback from the environment. Therefore, they tend to use avoidance and reflection when choosing coping strategies, which can easily lead to adverse adaptation [26].

To sum up, CT may influence depression via cognitive emotional regulation. Some existing studies have also investigated the mediated role of cognitive emotional regulation between childhood trauma and subsequent mental health problems. Both clinical sample study [12, 27] and college student sample study [28] provide evidence for that the negative effects of childhood trauma on later depression are mediated by emotion regulation. Furthermore, some other studies on children and adolescents [29] [30] have shown the mediator of emotion regulation between childhood trauma and psychopathology. As we have seen, the role of CERS as a mediator between CT and current depression symptoms have been supported by many findings.

#### Personality vulnerability

Zuroff proposed the interaction model of personality vulnerability to depression [31], which pointed out that the interaction between individual personality vulnerability and external stress events can affect depression and cause its exacerbation or persistence. Meanwhile, many studies have focused on the influence of personality traits between CT and depression. Lee and Hovens’ researches have shown that the personality traits, in particular neuroticism and extroversion, mediated the relationships between CT and depression in nonclinical or clinical patients [32, 33]. Likewise, a previous study based on longitudinal population-based twin cohort has revealed that compared to extraversion, levels of neuroticism more strongly predicted the risks for both lifetime and new-onset major depression [34]. Another cross-sectional study based on a sample of adults further revealed that only emotional stability (neuroticism) significantly mediated the influence of childhood abuse on depressive symptoms [32]. Among several personality traits, neuroticism plays a particularly prominent role.

However, besides mediated effect, some studies have pointed out that the neuroticism moderated the relationship between childhood trauma and depression. A study of internet addiction mediating the relationship between childhood trauma and depression among Chinese colleges, suggested that neuroticism moderated the direct path of childhood trauma on depression and the indirect path of internet addiction on depression [35, 36]. Ling Yu [37] and Xi Chang et al. [38] found that neuroticism plays a role in moderating the relationship between stress and depression through longitudinal studies and cross-sectional among Chinese adolescents and college students.

In addition, individuals with neurotic personality are more likely to have negative emotions (such as anxiety, depression, etc.) [39] and the neuroticism is closely related to CERS. Some studies have found that neuroticism is negatively correlated with adaptive CERS and positively correlated with maladaptive CERS in adolescents [40] and adults [41]. Furthermore, those who were exposed to CT had higher levels of neuroticism, which further contributed to depressive symptoms in adulthood [32].

### The current study

Although the relationship between CT and depression has been repeatedly verified by large number of studies, the mechanism of how CT affects depression is still unclear. Studies have verified the mediating role of CERS in the relationship between CT and depression in depressive patients [27], college students [28], adolescents [42] and other groups. Moreover, in previous studies, the role of neuroticism is somewhat inconsistent — mediator or moderator. Childhood trauma may affect current depressive symptoms through CERS, and what role does neuroticism play in this process? Under what circumstances does neuroticism affect the relationship between childhood trauma and depression?

Epidemiological studies have consistently found the high prevalence of many common mental disorders, especially the mood, anxiety and substance use disorders in university students [43, 44]. And the college years straddle a distinct development period of the adolescent and young adulthood life stages, charactered by sexual maturity, touched a range of educational and occupational opportunities, and substantial instability (changes in romantic status, peer groups, course selection, career choices et al) [45]. Therefore, the college stage represents a unique period. This study intends to integrate cognitive vulnerability (cognitive emotion regulation strategies) and personality vulnerability (neuroticism), in order to explore the psychological mechanism of the impact of CT on current depression in university students. In this way, it helps to clarify the research on the mechanism of childhood trauma affecting depression in university students. Moreover, it can also provide intervention guidance for promoting the mental health of university students and prevent future psychological problems.

As a result, the first hypothesis of this research is: adaptive and maladaptive CERS mediate the relationship between CT and current depression. CT may interact with neuroticism and affect depression. Accordingly, we proposed the second hypothesis: neuroticism may function as a moderator between CT and current depressive symptoms either separately, or both in the direct effect (CT → current depression) and indirect effect (path a1: CT → adaptive CERS, path a2: CT → maladaptive and path b1: adaptive CERS → current depression, path b2: maladaptive CERS → current depression).

**Fig. 1:**
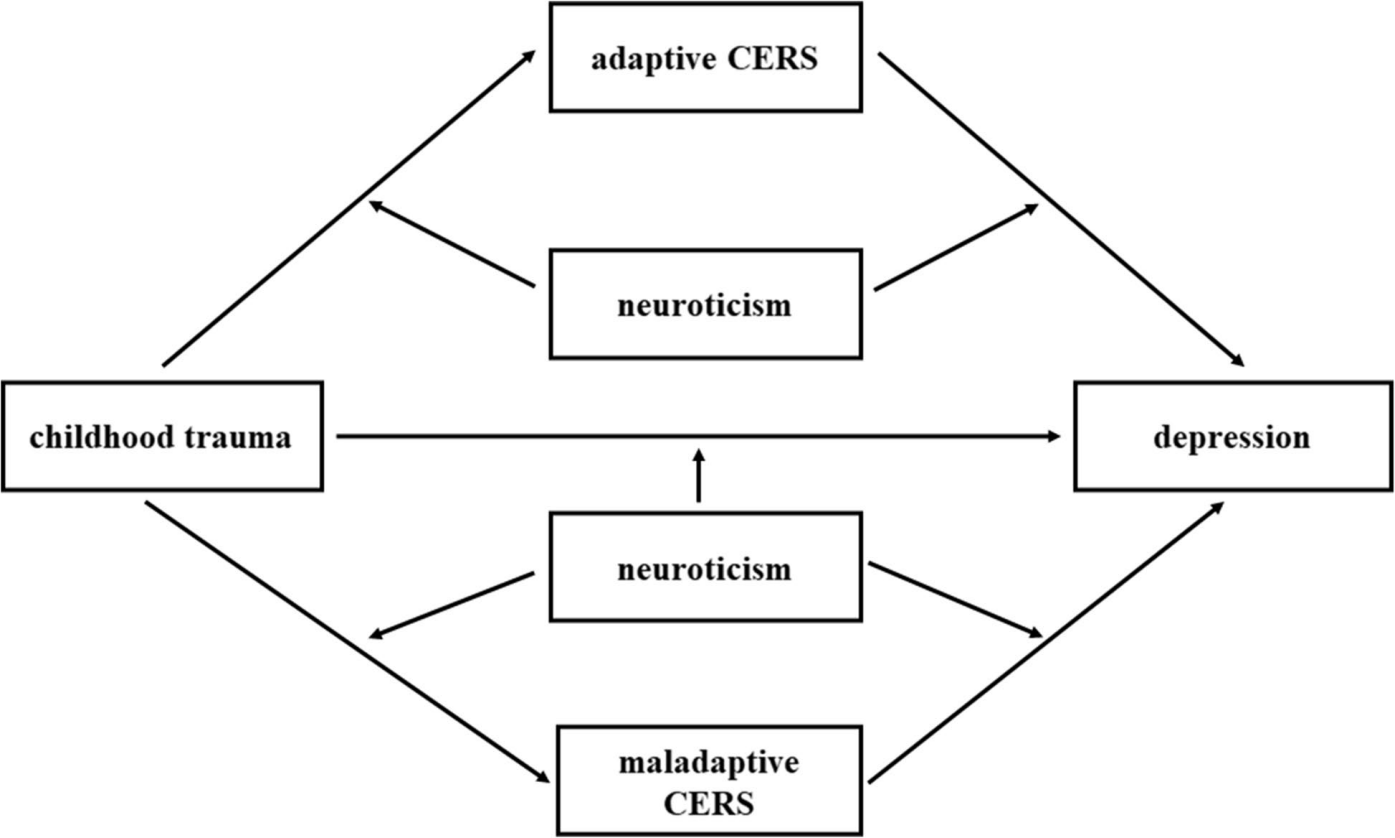
The proposed model. *Note*: CERS, cognitive emotion regulation strategies

We aim to examine the conceptual model (Fig. 1) in current study, in which, first, adaptive CERS and maladaptive CERS mediated the relationship between CT and current depression; second, the direct and indirect relationships between CT and current depression were moderated by neuroticism. Vulnerability and stress are important concepts in the vulnerability-stress model. Recent life stress usually acts as a trigger and activates the vulnerability, which in turn brought about the onset of depression. Thus, in order to clarify the impact of CT on current depression, we controlled the negative life events over the last 12 months as a covariable. Furthermore, we also controlled age, gender in this study. This research can help clarify the psychological mechanism of CT on depression, provide theoretical guidance for clinical intervention, and promote the mental health of university students.

## Materials and methods

### Participants

We conducted a large sample questionnaire survey for all freshmen (about 3500 students) through convenient sampling in a university in Hunan from October 27th to November 3rd, 2019. All participants were tested during evening self-study time, in exchange for no rewards or course credit. And all of them were given informed consent before completing the measures. Ultimately, 3009 participants (1014 men and 1995 women), mean aged 18 years (SD = 0.772) provided fully completed questionnaires with no missing data, which gave a response rate of 85.97%. The Ethics Committee of the Third Xiangya Hospital of Central South University approved this study.

### Measures

#### Childhood trauma

The Childhood Trauma Questionnaire (CTQ) [46], including 28 items self-report inventory, was used to evaluate five types of CT experience: emotional abuse, physical abuse, sexual abuse, emotional neglect and physical neglect. Each item uses a 5-point frequency scale (1 = never to 5 = always). The higher the total score means that the individual may suffer more severe trauma during childhood. So far, CTQ has been introduced in many countries and applied to different groups of people in the world, such as Norwegians in high-risk groups [47], both clinical and nonclinical samples in Swedish [48] and middle school students in China [49]. And all of them have reported a good internal consistency. In this study, the CTQ total score also displayed an acceptable internal consistency (Cronbach’s α = 0.662).

#### Depressive symptoms

The Beck Depression Inventory-II (BDI-II) [50] as a 21-item self-report measure was used to evaluate depression severity. Each item was rated from 0 to 3 and the total scores range from 0 to 63. Furthermore, according to the final scores, depressive symptoms can be categorized into four levels: minimal depression (total score, 0–13); mild depression (total score, 14–19); moderate depression (total score, 20–28); severe depression (total score, 29–63). The Chinese version of BDI-II translated by Yang Wen-hui [51] was adopted in this study. The Chinese versions of the BDI-II [51] have been well validated. In this study, BDI-II (Cronbach’s α = 0.907) also showed good internal consistency.

#### Adaptive / maladaptive cognitive emotion regulation strategies (CERS)

Cognitive emotion regulation strategies which people tend to choose when they encounter negative life events, were assessed by the Cognitive Emotion Regulation Questionnaire (CERQ) [16, 52]. This is a 36-item questionnaire, adopting 5-point Likert scale (1 = almost never to 5 = almost always). There are nine subscales in this questionnaire, and each subscale contains four items. Nine subscales represent nine cognitive emotion regulation strategies respectively: rumination, catastrophizing, self-blame, blaming others, putting into perspective, acceptance, positive refocusing, positive reappraisal, and refocusing on planning. The self-blame, rumination, catastrophizing, and blaming others are regarded as maladaptive, whereas acceptance, positive refocusing, refocus on planning, positive reappraisal, and putting into perspective are regarded as adaptive. Moreover, the higher score means that the person uses the strategy more frequently in response to a negative event. CERQ has been validated for different languages and populations, for instance: adolescents aged 14–18 [53] and older aged adults 65–90 [54] in Spanish, university students in Brazilian [55], and adults with recurrent depression [56]. All of them have proved that the CERQ is a valid and reliable tool for assessing cognitive emotion regulation strategies. The Chinese version of CERQ [57] was used in this study. Cronbach’s α for nine subscales were ranged 0.586–0.912, and the full scale’s Cronbach’s α was 0.875 in current research.

#### Neuroticism

Eysenck Personality Questionnaire (EPQ) was developed by Eysenck and colleagues, as a measure of personality traits [58]. G,Y.X [59] revised the Chinese version of EPQ in 1992, which consists of 88 items, and included four subscales: extroversion (E), neuroticism (N), psychoticism (P) and lie (L), where neuroticism(N) represented emotional instability and anxiousness. We adopted the Neuroticism Subscale of Eysenck’s Personality Questionnaire (EPQ-N) to assess the neuroticism in present study. EPQ-N consists of 24 items, and each item is responded to using “yes” or “no” (yes = 1, no = 0). Higher scores reflect higher level of neuroticism, which means that individuals are more likely to experience some negative emotions, such as moodiness, worry and anxiety. Cronbach’s α of EPQ-N was 0.895 in this study.

#### Negative life events

The current study adopted the Adolescent Self-Rating Life Events Check (ASLEC) to assess negative life events during the past 12 months. It contains of the 27 common stressful life events, summarized by the following 6 factors: interpersonal relationship, academic stress, punishment, loss, health adaptation and other factors [60]. The participants first identified if the event had occurred in the past 12 months. If it had, they were required to rate on 5-point scale according to their psychological feelings. (1 = no influence, 2 = mild influence, 3 = moderate influence, 4 = severe influence, and 5 = extremely severe influence). If the event didn’t happen, it’s deemed to have no influence. By calculating the scores of all items, we can get a total life stress score. Higher score indicates higher influence of negative life events. ASLEC was reported a good reliability and validity in many studies [60, 61], the Cronbach’s α in current study was 0.900.

### Statistical analyses

Descriptive analyses were conducted by SPSS 23.0. Research variables (CT, depression, adaptive / maladaptive cognitive emotional regulation strategies, neuroticism, negative life events) were analyzed by Pearson correlation analyses. The moderated mediation analysis was conducted by using the SPSS PROCESS 3.5 macro [62], for the purpose of testing the hypothesized model (Fig. 1). Firstly, we tested whether the association between CT and depressive symptoms was mediated by adaptive and maladaptive CERS using Model 4. Next, Model 59 was used to examine the moderated mediation effect. And we chose non-parametric percentile bootstrap method for bias correction. We conduct bootstrapping of regression estimates with 5000 samples and 95% confidence interval. If the confidence interval did not include 0, the effect is considered significant. The grouping conditions were set to mean and mean ± 1 SD [63]. In addition, all models were controlled for covariates (age, sex, negative life events).

## Results

### Preliminary analysis

The present study recruited 3009 subjects from university students in Hunan Province. In this study sample, a total of 371 (12.33%) participants were found to have depressive symptoms: 214 (7.11%) participants belong to mild depression; 121 (4.02%) participants belong to moderate depression; and 36 (1.20%) participants belong to severe depression. A total of 943 subjects (31.34%) reported experiencing CT. To be more specific, 620 (20.60%) subjects reported experiencing physical neglect; 376 (12.59%) subjects reported experiencing emotional neglect; 126 (4.19%) subjects reported experiencing physical abuse; 121 (4.02%) subjects reported experiencing emotional abuse; 184 (6.11%) subjects reported experiencing sexual abuse. This information is shown in Table 1.

**Table 1:** Socio-demographic characteristics of participants (*N* = 3009)

Characteristic	Mean (SD) / N (%)
Age	18.00 (0.772)
Gender male	1014 (33.70%)
female	1995 (66.30%)
Main place of residence	
urban	1299 (43.17%)
rural	1549 (51.48%)
missing	161 (5.35%)
Depressive symptoms	371 (12.33%)
mild depression	214 (7.11%)
moderate depression	121 (4.02%)
severe depression	36 (1.20%)
Childhood trauma	943 (31.34%)
physical neglect	620 (20.60%)
emotional neglect	376 (12.59%)
physical abuse	126 (4.19%)
emotional abuse	121 (4.02%)
sexual abuse	184 (6.11%)

*Note: SD*, standard deviation

Means, SDs and correlation matrix for several crucial research variables are presented in Table 2**.** CT was positively correlated with depression (*r* = 0.339, *p* < 0.01), neuroticism (*r* = 0.321, *p* < 0.01), maladaptive CERS (*r* = 0.199, *p* < 0.01). Also, CT was negatively correlated with adaptive CERS(*r* = − 0.120, *p*<0.01). Adaptive CERS was not significantly correlated with neuroticism, negative life events, and depression. Maladaptive CERS was positively correlated with depression (*r* = 0.408, *p* < 0.01), negative life events(*r* = 0.359, *p*<0.01). Neuroticism was positively correlated with depression (*r* = 0.654, *p* < 0.01), negative life events (*r* = 0.470, *p* < 0.01). As we expected, university students with higher CT had more use of maladaptive CERS and more severe depression in adulthood.

**Table 2:** Descriptive statistics and related analysis results of major variables

	1	2	3	4	5	6
1. CT	1	− 0.120**	0.199**	0.321**	0.339**	0.317**
2. adaptive CERS		1	0.304**	0.018	−0.009	0.014
3. maladaptive CERS			1	0.534**	0.408**	0.359**
4. neuroticism				1	0.654**	0.470**
5. depression					1	0.440**
6. negative life events						1
M	45.609	66.379	40.938	9.569	6.060	50.637
SD	7.459	9.513	7.697	5.919	6.735	15.584

*Note*: N = 3009; significance was set as *p*<0.01; ** *p*<0.01

*Abbreviations: M*, mean scores; *SD*, standard deviation; *CT*, childhood trauma; *CERS*, cognitive emotion regulation strategies

### Mediation analyses

Table 3 showed the results of mediation. The total effect of CT on current depression is estimated as c = 0.206 (95% CI: 0.176 to 0.236), and the direct effect is estimated as c′ = 0.170 (95% CI: 0.141 to 0.199). The paths from CT to adaptive CERS (*β* = −0.141, 95% CI: − 0.227 to − 0.131) and maladaptive CERS (*β* = 0.093, 95% CI: 0.059 to 0.132) were both significant. Also, the paths of from adaptive CERS to current depression (*β* = − 0.084, 95% CI: − 0.082 to − 0.037) and from maladaptive CERS to current depression (*β* = − 0.299, 95% CI: 0.233 to 0.292) were also significant. The bootstrapping index for an indirect effect (*β* = 0.012) was significant when adaptive CERS was included as mediating variables since the 95% confidence interval does not include zero (0.006 to 0.018). the bootstrapping index for indirect effect of maladaptive CERS was also significant (*β* = 0.028,95% CI: 0.016 to 0.040. Therefore, the mediating effect of adaptive CERS and maladaptive CERS on the relationship between CT and current depression were significant. The indirect effect of CT on current depression through adaptive CERS accounted for 5.69% of the total effect. And the indirect effect of CT on depression current through maladaptive CERS accounted for 13.52% of the total effect.

**Table 3:** Mediation effect of CERS (by parallel mediation analysis)

Outcome Variables	Adaptive CERS			Maladaptive CERS			Depression		
	*β*	LCI	UCI	*β*	LCI	UCI	*β*	LCI	UCI
CT	-0.141**	-0.227	-0.131	0.093**	0.059	0.132	0.188**	0.141	0.199
Adaptive CERS							-0.084**	-0.082	-0.037
Maladaptive CERS							0.299**	0.233	0.293

Note: significance was set as *p *< 0.05; ** *p *<0.01, * *p *<0.05

*Abbreviations:* *β*, standardized regression coefficient; *CT* childhood trauma; *CERS* cognitive emotion regulation strategies; *LCI* lower bound of 95% confidence interval; *UCI* upper bound of 95% confidence interval

Then we explored the mediation role of specific types of CERS in the relationship between CT and current depression, conducting follow-up parallel mediation analysis. Parallel mediation analysis results showed that mediating effects of acceptance (*β* = − 0.004, 95% CI: − 0.008 to − 0.001), positive refocusing (*β* = 0.004, 95% CI: 0.001 to 0.009), positive reappraisal (*β* = 0.032, 95% CI: 0.021 to 0.044), putting into perspective (*β* = 0.003,95% CI: 0.0001 to 0.007), blame-others (*β* = − 0.004, 95% CI: − 0.008 to − 0.001) and catastrophizing (*β* = 0.034, 95% CI: 0.024 to 0.046) were significant. However, for the remaining types of CERS, the mediating effects were not significant.

### Moderated mediation analyses

The results of moderated mediation analyses (Table 4) showed that neuroticism simultaneously moderated the direct effect of CT on current depression (*β* = 0.035, 95% CI: 0.001 to 0.009), and the indirect effects of CT on current depression through adaptive CERS (adaptive CERS - current depression: *β* = − 0.034, 95% CI: − 0.007 to − 0.001) and maladaptive CERS (maladaptive CERS - current depression: *β* = 0.157, 95% CI: 0.017 to 0.025). However, the moderating effects of neuroticism were not significant in the indirect paths from CT to adaptive CERS (*β* = 0.037, 95% CI: 0.000 to 0.014) and maladaptive CERS (*β* = − 0.001, 95% CI: − 0.006 to 0.005). The moderated mediation results of specific adaptive CERS (acceptance, positive refocusing, positive reappraisal, putting into perspective) and maladaptive CERS (blame-others, catastrophizing) showed by additional file 1.

**Table 4:** Moderated mediation analysis results for the relationship between CT and current depression

	M1			M2			Depression (Y)		
	*β*	LCI	UCI	*β*	LCI	UCI	*β*	LCI	UCI
CT (X)	−0.155**	−0.227	− 0.131	0.093**	0.059	0.132	0.089**	0.053	0.108
Adaptive CERS (M1)							−0.049**	−0.054	− 0.015
Maladaptive CERS (M2)							0.084**	0.045	0.103
Neuroticism (W)							0.494**	0.524	0.601
CT × neuroticism	0.037	0.000	0.014	−0.001	−0.006	0.005	0.035*	0.001	0.009
Adaptive CERS × neuroticism							−0.034*	−0.007	−0.001
Maladaptive CERS × neuroticism							0.157**	0.017	0.025
	R^2^ = 0.022			R^2^ = 0.139			R^2^ = 0.491		
F	13.157**			96.030**			287.622**		

*Note*: significance was set as *p* < 0.05; ** *p*<0.01, *p<0.05

Abbreviations: *β*, standardized regression coefficient; *CT*, childhood trauma; *CERS*, cognitive emotion regulation strategies; *LCI*, lower bound of 95% confidence interval; *UCI*, upper bound of 95% confidence interval

**Fig. 2:**
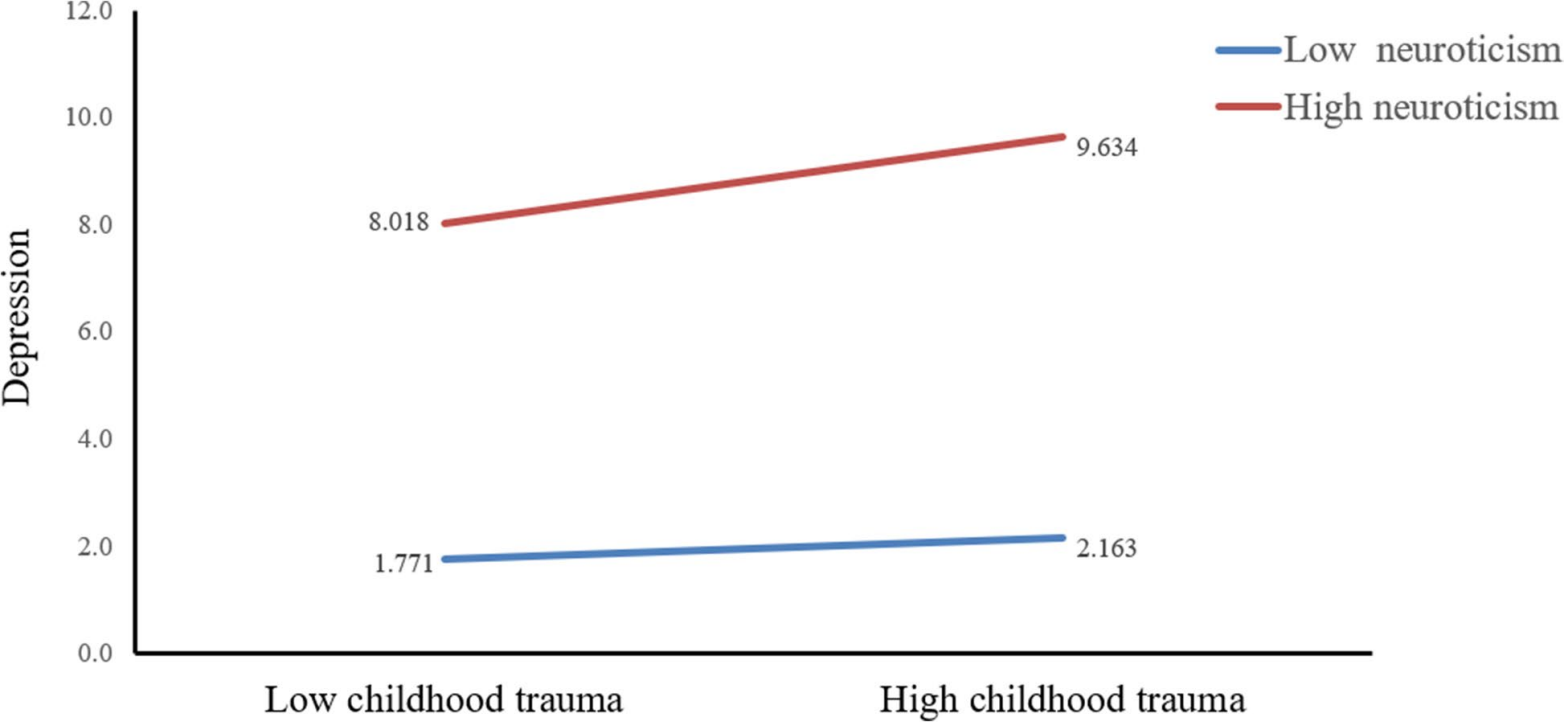
The conditional effect of CT on depression at the values of neuroticism. *Note*: CT, childhood trauma

**Fig. 3:**
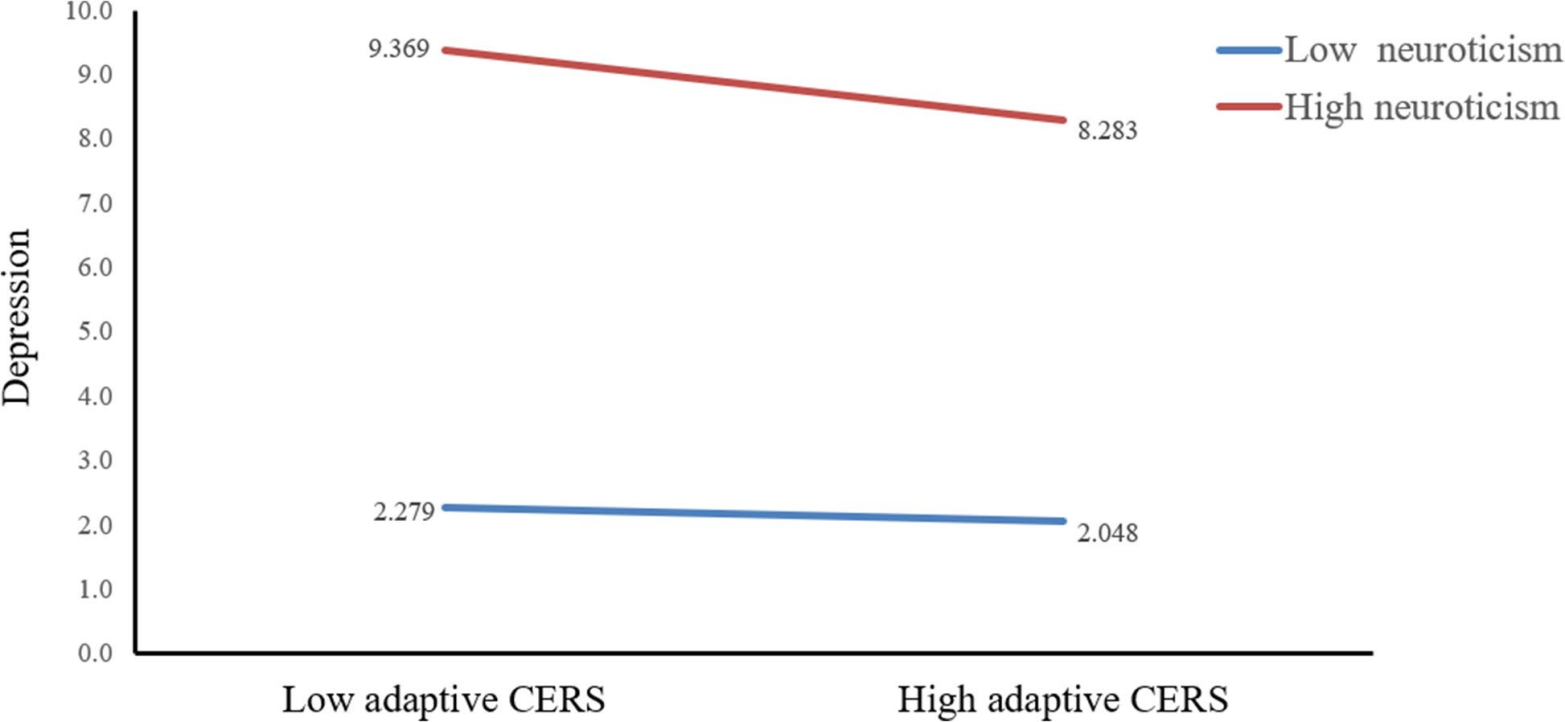
The conditional effect of adaptive CERS on depression at the values of neuroticism. *Note*: CERS, cognitive emotion regulation strategies

**Fig. 4:**
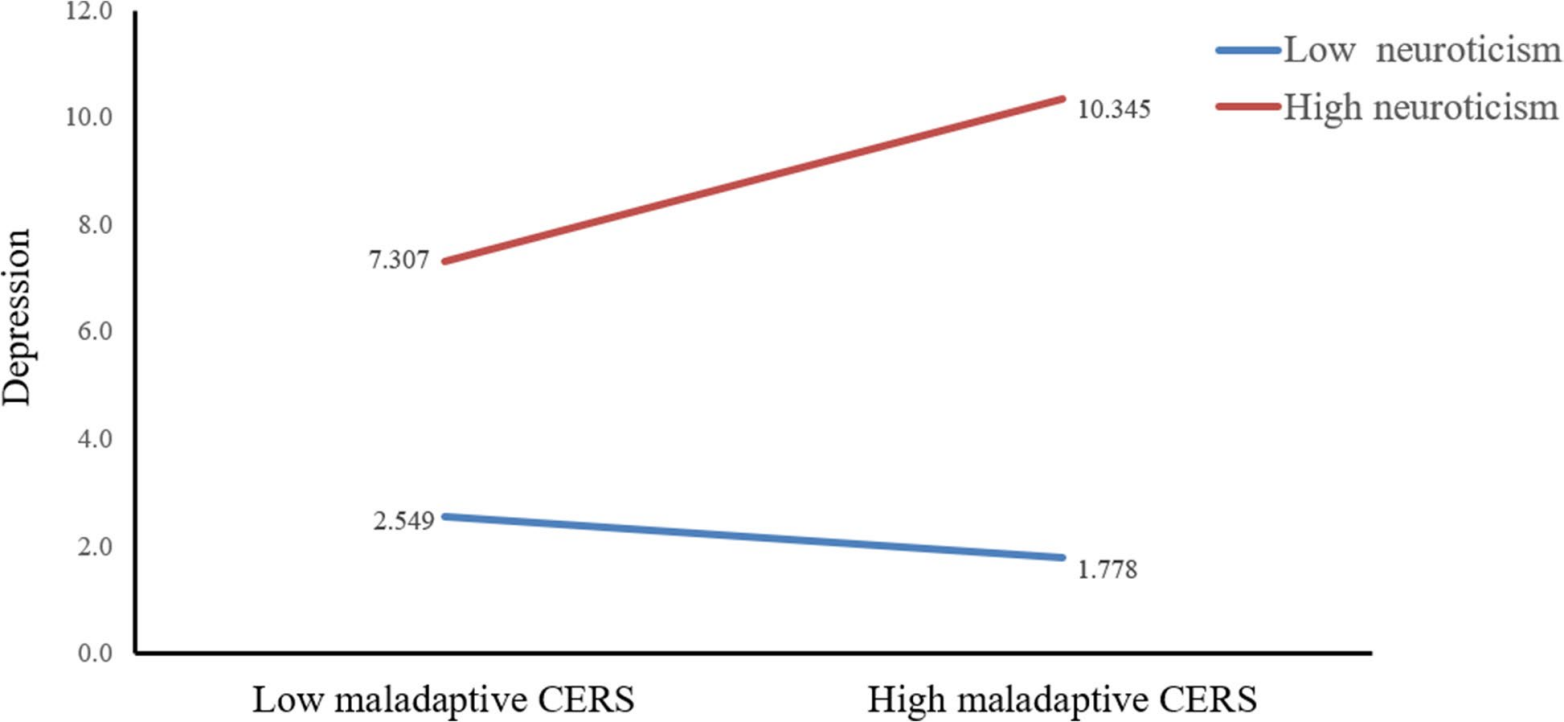
The conditional effect of maladaptive CERS on depression at the values of neuroticism. *Note*: CERS, cognitive emotion regulation strategies

**Fig. 5:**
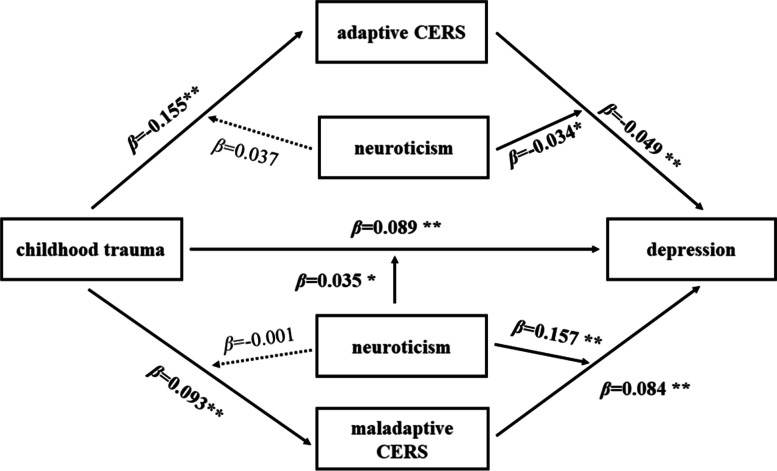
**The final moderated mediation model.** *Note*s: significance was set as *p*<0.05; ^**^ *p*<0.01, ^*^ *p*<0.05. Abbreviation: CERS, cognitive emotion regulation strategies

In order to further analyze the direct effect and indirect effects of CT on current depression at different levels of neuroticism, we divided neuroticism into three levels of low, medium, and high according to M - SD, M and M + SD. The results are shown as Table 5**.** Specifically, the direct impact of CT on depression were all significant in low-neuroticism (*B* = 0.053, 95% CI: 0.011 to 0.095), in medium-neuroticism (*B* = 0.081, 95% CI: 0.053 to 0.108) and high-neuroticism (*B* = 0.109, 95% CI: 0.080 to 0.137) (see Fig. 2). In addition, the indirect impact of adaptive CERS on depression were significant when neuroticism for individual was medium (*B* = − 0.35, 95% CI: − 0.055 to − 0.015) and high (*B* = − 0.057, 95% CI: − 0.086 to − 0.029). However, when the level of neuroticism was low, the influence of adaptive CERS on depression was not significant (see Fig. 3). Fig. 4 showed that neuroticism could moderate the association between maladaptive CERS and current depression at any value: Low-neuroticism (*B* = − 0.050, 95% CI: − 0.086 to − 0.015), medium-neuroticism (*B* = 0.074, 95% CI: 0.045 to 0.103) and high-neuroticism (*B* = 0.198, 95% CI: 0.159 to 0.236). The final moderated mediation model was displayed in Fig. 5

**Table 5:** Conditional direct and indirect effects of CT on depression at different values of neuroticism

	CT → Depression			Adaptive CERS → Depression			Maladaptive CERS → Depression		
	*B*	LCI	UCI	*B*	LCI	UCI	*B*	LCI	UCI
Low neuroticism(−SD)	0.053*	0.011	0.095	−0.012	−0.039	0.014	−0.050 **	− 0.086	−0.015
Medium neuroticism (SD)	0.081 **	0.053	0.108	−0.035**	−0.055	− 0.015	0.074*	0.045	0.103
High neuroticism (+SD)	0.109 **	0.080	0.137	−0.057**	−0.086	− 0.029	0.198 **	0.159	0.236

*Note*: significance was set as *p* < 0.05; ** *p*<0.01, * *p*<0.05

Abbreviations: *B*, unstandardized regression coefficient; *CT*, childhood trauma; *CERS*, cognitive emotion regulation strategies; *LCI*, lower bound of 95% confidence interval; *UCI*, upper bound of 95% confidence interval

## Discussion

In current study, we tested the hypothesis with moderated mediation model in which CT was associated with current depression as mediated by CERS (adaptive and maladaptive CERS) and moderated by neuroticism **(**Fig. 1).

### The mediating role of adaptive and maladaptive CERS

Consistent with our hypothesis 1, mediation analyses results showed that the adaptive CERS and maladaptive CERS partially mediated the relationship between childhood traumatic experience and current symptoms of depression.

CT was negatively correlated with adaptive CERS and positively correlated with maladaptive CERS. Some researchers pointed out that CT may cause emotion dysregulation in later life [64, 65]. And failing to regulate the emotion is closely related to the occurrence of psychological problems [21]. Our mediation analysis results showed that the maladaptive CERS significantly mediated the relationship between CT and current depression, which confirmed that CT could affect current depression through using maladaptive CERS. Our results also revealed the significant mediating effect of adaptive CERS, and highlighted the importance of using adaptive CERS. Namely, using adaptive CERS may weaken the influence of CT on current depression. This finding is consistent with previous research [28], which acknowledged that the use of cognitive emotion regulation strategies is an important possible mechanism underlying the negative effect of CT on depressive severity in later life. However, some previous studies have shown that the mediating effect of adaptive CERS was not significant [12, 66]. They believed the indirect effect of adaptive CERS was weaker than maladaptive CERS. The reason for this inconsistent result may be caused by using healthy university students in this study whose depression symptoms are generally milder than those in the clinical group. Clinical groups may have difficulty in implementing adaptive strategies effectively compared with non-clinical groups [15]. Therefore, for non-clinical groups especially university students, the adaptive CERS may show stronger indirect effects among the CT and current depressive symptom.

In our results, the mediating effects of acceptance, positive refocusing, positive reappraisal, putting into perspective, blame-others and catastrophizing were significant, and the mediating effects of the remaining CERS types were not significant. Regarding the mediating role of specific cognitive emotion regulation strategies, the results of many studies are not completely consistent [27, 28, 42]. Aldao and Nolen-Hoeksema [67] found that the degree to which reappraisal is adaptive depends on the frequency of maladaptive strategy used. This may also be one of the reasons why the results of the specific CERS analysis in this study are different from previous studies. Moreover, we have not considered that the frequency of using maladaptive CERS would influence the effect of adaptive CERS. To further investigate the specific CERS’s exact effect in the relationship between early traumatic experience and psychiatric symptoms, more research is required in this field.

### The moderating role of neuroticism

Moderated mediation analysis largely supports our research hypothesis. It demonstrated that CT can affect the occurrence and development of depression through using adaptive / maladaptive CRES, and neuroticism plays a moderator role in the direct impact of CT on depression, and the indirect impacts from adaptive and maladaptive to depression.

Through simple slope analysis, we found that compared with low neurotic individuals, high neurotic individuals have a more pronounced increase in the severity of depression as the level of CT increased (Fig. 2). Consistent with one study among Chinese university students, neuroticism moderated the relationship between stress and depression [38]. In the process of CT affecting depression, neuroticism, as an essentially vulnerable factor, interacted with various stressors [31]. For that reason, using adaptive CERS has an obvious reduction effect on depression in high neurotic people than in low neurotic people (Fig. 3). Some researchers pointed out that more use of adaptive CERS (e.g. refocusing on planning) is beneficial to resilience and depression [68]. The protective effect of adaptive CERS on depression is more pronounced in highly neurotic people. Then, the depressive severity in the low-neuroticism group decreased with the increase of maladaptive CERS, whereas depressive severity in the high-neuroticism group increased obviously. In other words, low-neuroticism can weaken the effect of maladaptive CERS on depression (Fig. 4). as such, maladaptive CERS was positively related to depressive symptoms. A study of using specific emotion regulation strategies in clinical patients suggested that use of maladaptive CERS was an universal feature of depression and anxiety disorders [69]. This research revealed that maladaptive CERS may increase the level of depression, and this effect is strengthened in people with high neuroticism, and low neuroticism may attenuate this effect.

In a nutshell, our findings broaden previous researches by exploring the mediating roles of adaptive CERS and the moderating role of neuroticism. This result validates the interaction model of personality vulnerability to depression [31]. Neuroticism, as an individual’s specific vulnerability quality, interacts with the experience of CT to affect the current depressive symptoms. Specifically, highly neurotic individuals exposed to childhood trauma are high-risk groups of depression and CERS intervention can be used for high-risk groups. In high-risk groups, adaptive CERS plays a protective role in the process of childhood trauma affecting depression, while maladaptive CERS plays a risk role. Furthermore, we also found that low neuroticism plays a protective role in the process of maladaptive CERS effecting depression. For that reason, the influence of neuroticism should be fully considered when intervening in CERS, so that a more appropriate interventional method can be selected and the effects can be estimated in an accurate manner.

## Strengths and limitations

This study validates a new moderated mediation model, demonstrating the moderating role of neuroticism, providing evidence for the mechanism of CT on depression, and correct theoretical guidance for our clinical intervention. In addition, we have controlled the impact of recent stressors, which makes our results more rigorous.

The study also has several limitations. First, this study assessed measurements concurrently as a cross-sectional study. However, in order to better determine the causality, it is necessary to conduct further longitudinal studies on children who experience early life trauma. Especially, the prospective studies about serially assessing changes in emotion regulation ability and mental health outcomes would be informative. Second, we did not investigate and discuss individual differences of using CERS. Individuals do not use a single cognitive emotional regulation strategy. The reality is usually a combination of several strategies. Third, this study was only for university students, and the sample representation was limited.

## Conclusion

This study provides powerful evidences through a large university students sample for the mediating role of adaptive / maladaptive cognitive emotion regulation strategies and the moderating role of neuroticism between CT and current depression. Specifically, CT can directly affect current depression, and it can also affect depression through adaptive CERS and maladaptive CERS. Furthermore, different levels of neuroticism would affect the impact of CT on current depression and the impacts of adaptive CERS and maladaptive CERS on current depression. This manifests that cognitive emotion regulation may be a vital factor for people who suffered from CT and current depression, and the influence of neuroticism in this process cannot be ignored.

## Supplementary Information

**Additional file 1.**

## Data Availability

The datasets used and analyzed during the current study are available from the corresponding author on reasonable request.

## Abbreviations

CT: Childhood trauma
CERS: Cognitive emotion regulation strategies
CTQ: Childhood Trauma Questionnaire
BDI-II: Beck Depression Inventory-II
CERQ: Cognitive Emotion Regulation Questionnaire
EPQ: Eysenck Personality Questionnaire
EPQ-N: Neuroticism Subscale of Eysenck’s Personality Questionnaire
ASLEC: Adolescent Self-Rating Life Events Check
SD: Standard Deviation
CI: Confidence Interval
UCI: Upper Confidence Interval
LCI: Lower Confidence Interval
SPSS: Statistical Package for Social Sciences
