# Correction: Childhood trauma and current depression among Chinese university students: a moderated mediation model of cognitive emotion regulation strategies and neuroticism

**DOI:** 10.1186/s12888-022-03960-w

**Published:** 2022-05-16

**Authors:** Qianqian Chu, Xiang Wang, Rui Yao, Jie Fan, Ya Li, Fei Nie, Lifeng Wang, Qiuping Tang

**Affiliations:** 1 Department of Clinical Psychology, The Third Xiangya Hospital of Central South University, Changsha, 410013 Hunan China; 2 Medical Psychological Center, The Second Xiangya Hospital of Central South University, Changsha, 41000 Hunan China; 3 Center for Psychological Development and Service, Hunan University of Chinese Medicine, Hunan, 410208 Changsha China; 4 School of Nursing, Hunan University of Chinese Medicine, Changsha, 410208 Hunan China

**Correction to: BMC Psychiatry 22, 90 (2022)**

**https://doi.org/10.1186/s12888-021-03673-6**

Following publication of the original article [1], the authors would like to correct the numbering of in-text citations.

The original article [1] has been corrected.
