# Supplementary material for: Childhood trauma and current depression among Chinese university students: a moderated mediation model of cognitive emotion regulation strategies and neuroticism
Source: BMC Psychiatry. 2022 Feb 7;22:90. doi: 10.1186/s12888-021-03673-6 (PMC8819909; doi:10.1186/s12888-021-03673-6)
Supplement: Supplementary file 1 — Additional file 1. [file 12888_2021_3673_MOESM1_ESM.pdf]

Additional file 1. Moderated mediation analysis results for the specific CERS.

	Adaptive CERS β (LCI- UCI)				Maladaptive CERS β (LCI- UCI)		Depression β (LCI- UCI)
	Acceptance	Positive refocusing	Positive reappraisal	Putting into perspective	Blame-others	Catastrophizing	
CT	-0.082** [-0.044, -0.014]	-0.101**[-0.056, -0.023]	-0.164** [-0.082, -0.049]	-0.010 [-0.016, 0.010]	0.030[-0.004, 0.027]	0.103** [0.027, 0.054]	0.086** [0.051, 0.105]
Acceptance							0.085** [0.147, 0.289]
Positive refocusing							-0.032* [-0.138, -0.009]
Positive reappraisal							-0.084** [-0.260, -0.123]
Putting into perspective							0.043** [0.038, 0.203]
Blame-others							-0.040* [-0.166, -0.022]
Catastrophizing							0.046** [0.025, 0.188]
Neuroticism	0.055* [0.006, 0.043]	0.056** [0.007, 0.048]	-0.167** [-0.104, -0.064]	0.266** [0.093, 0.125]	0.286** [0.121, 0.159]	0.420**[0.189, 0.224]	0.491** [0.521, 0.596]
CT × neuroticism	-0.001[-0.002,0.002]	0.023[-0.001, 0.004]	0.050** [0.001, 0.005]	-0.001[-0.002, 0.002]	-0.021[-0.003, 0.001]	0.001[-0.002, 0.002]	0.034**[0.001, 0.008]
Acceptance × neuroticism							0.103** [0.030, 0.052]
Positive refocusing × neuroticism							-0.024[-0.019, 0.002]
Positive reappraisal × neuroticism							-0.057** [-0.032, -0.010]
Putting into perspective × neuroticism							0.020 [-0.005, 0.023]
Blame-							-0.037** [-0.026, -0.002]

others × neuroticism							
Catastrophizing × neuroticism							0.131** [0.033, 0.059]
	R^2=0.011	R^2=0.010	R^2=0.062	R^2=0.091	R^2=0.134	R^2=0.279	R^2=0.512
**F**	5.586**	4.960**	32.736**	49.472**	77.116 **	192.297 **	173.111**

Notes: ** $P < 0.01$, * $P < 0.05$

Abbreviations: CERS, cognitive emotion regulation strategies; CT, childhood trauma; β, standardized regression coefficient; LCI, lower bound of 95% confidence interval; UCI, upper bound of 95% confidence interval.
